# Cherubism: Cone-Beam Computed Tomography Findings in Two Siblings

**DOI:** 10.7759/cureus.54465

**Published:** 2024-02-19

**Authors:** Swapnil Mohod, Komal V Dadgal

**Affiliations:** 1 Oral Medicine and Radiology, Sharad Pawar Dental College and Hospital, Datta Meghe Institue of Higher Education and Research, Wardha, IND

**Keywords:** nonneoplastic condition, radiographic investigations, cbct, multilocular lesion, jaw disease, cherubism, familial disease

## Abstract

Cherubism is an uncommon, multilocular cystic condition of the jaws. This autosomal dominant, nonneoplastic, fibro-osseous condition of the jaws affects children and is self-limiting. Clinically, it shows up as an expansion or distortion of the jaw together with an unnatural teeth alignment. In their most severe forms, these deformations might cause phonation difficulties, decreased pharynx lumen, visual issues, and psychological effects. This case report describes a case report of two siblings involving the maxilla and the mandible presenting with a painless bilateral symmetrical enlargement. Cherubism is a hereditary condition characterized by nonneoplastic bone lesions that damage the jaws. Surgery should be used to resolve any functional or aesthetically problematic issues. Typically, the surgical procedure is postponed until after puberty.

## Introduction

Cherubism has long been misinterpreted as a hereditary type of fibrous dysplasia since it was first identified by Dr. W. A. Jones of Kingston, Ontario in 1933. The mandibular and maxillary bones are the only body parts affected by this uncommon, hereditary autosomal dominant condition. Because of the patients' angelic features (chubbiness and an upward gaze), cherubism got its name, according to Jones (1933) [1]. It is widely believed to be a benign, genetic bone condition that starts at two or three years old, progresses throughout childhood, peaks around age five, and then spontaneously regresses toward the end of puberty.

Seward and Hankey in 1957 proposed a cherubism grading system. Grade I: Involvement of the body of mandible, molar areas on each side, ascending rami, or symphyseal region. Grade II: Widespread mandibular involvement and maxillary tuberosities bilaterally (with grade I lesions). Grade III: Exception to the condyles, there is extensive involvement of both the jaws. Grade IV: Both maxilla and mandible are affected, including the condyles [1].

## Case presentation

Two sisters aged eight years (Patient 1) and 10 years (Patient 2) reported a complaint to the outpatient department alleging bilateral, painless swelling of the maxilla and the mandible. The parents reported that the swelling of the younger and elder daughter’s jaws has been noticeable for three years and five years, respectively, which has gradually progressed to the current size. Other than that, the children had no relevant medical history and were in good health. There was no history of facial defects in other family members. Physical and mental development was normal.

On physical examination, it was seen that both patients were cooperative, conscious, and well-oriented to time, place, and person. Clinical examinations of the chest, abdominal region, cardiovascular, and central nervous system revealed no abnormalities. There were no congenital abnormalities or cutaneous pigmentation.

**Figure 1:**
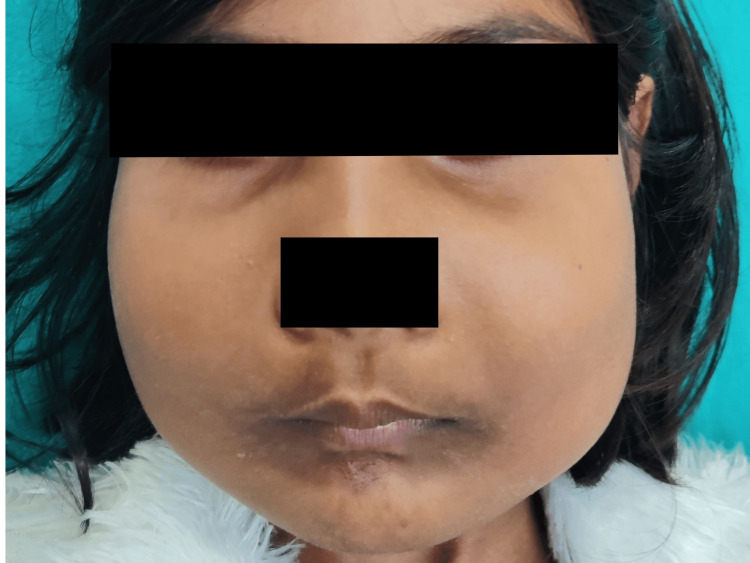
(Patient 1: eight years) Photograph of the patient showing the frontal view of the face with symmetrical bilateral swelling

**Figure 2:**
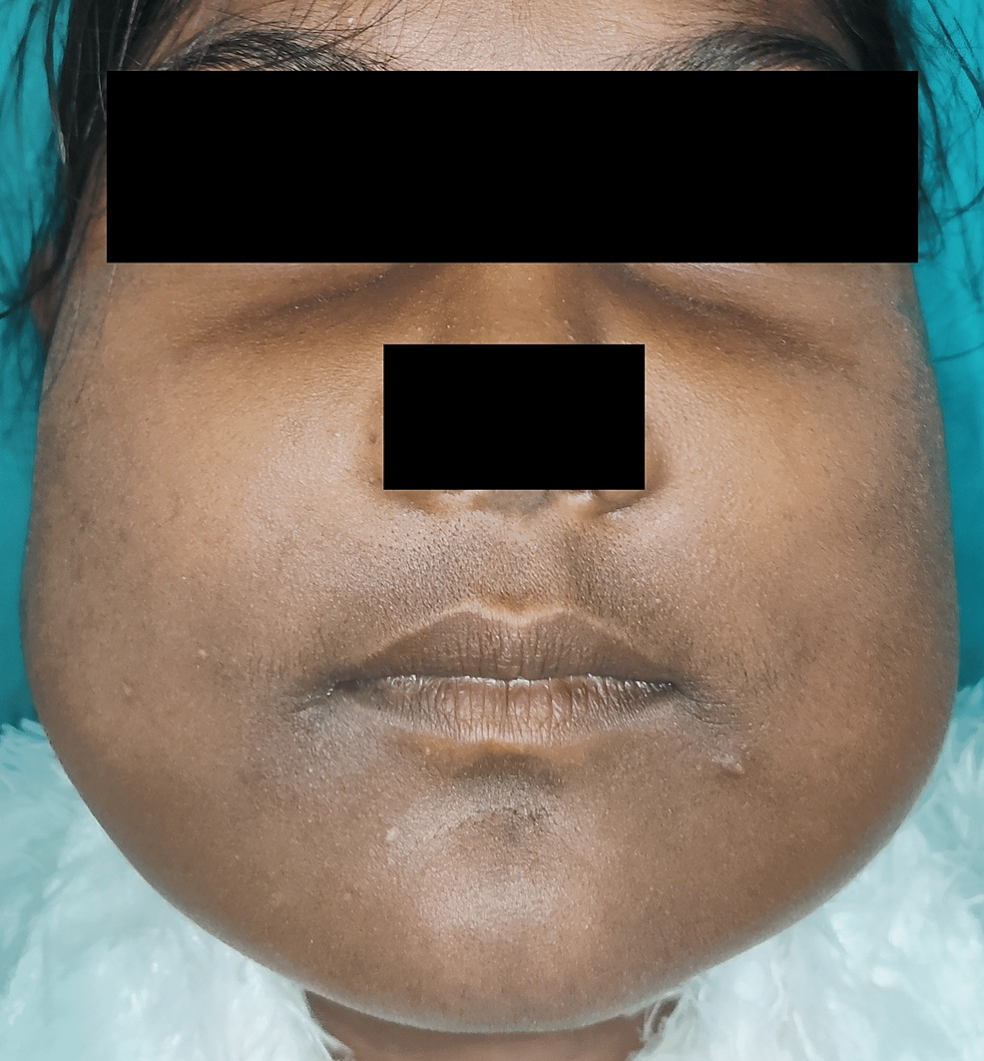
(Patient 2: 10 years) Photograph of the patient showing the frontal view of the face with symmetrical bilateral swelling

Extraoral examination revealed bilateral swelling involving the maxilla and mandible. The margins of the swelling were diffused, and the swelling's surface was smooth. The swollen skin was the same as the adjacent skin with no visible pulsations. Upon palpation, the swelling was firm and nontender, and the local temperature had not increased. In both children, bilateral submandibular lymph nodes were nontender, firm, and mobile on palpation (Figure 1 and Figure 2).

**Figure 3:**
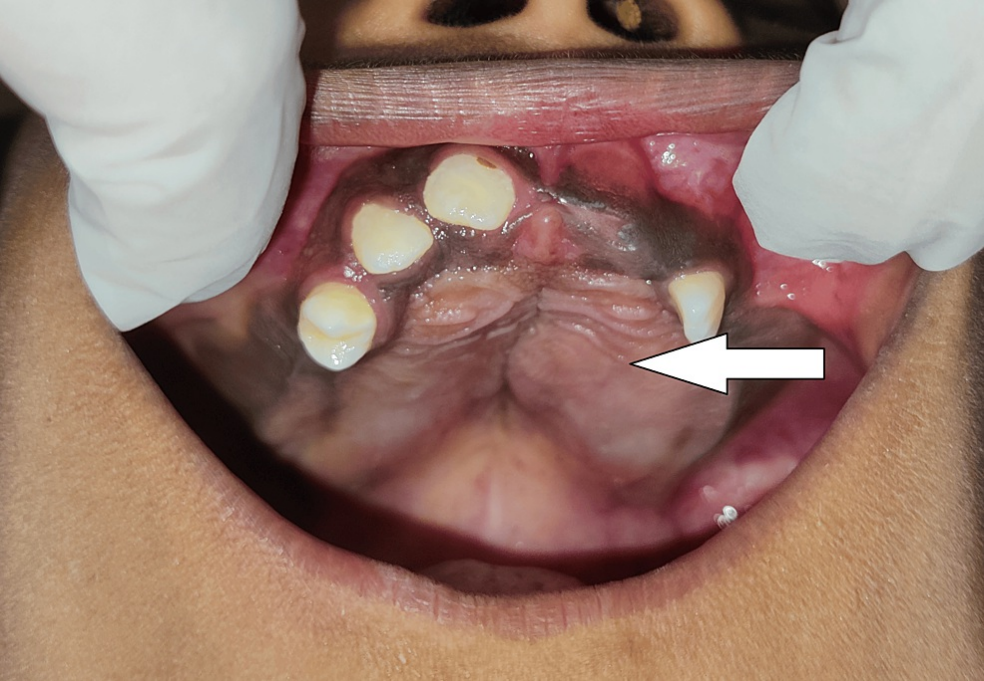
Patient 1 younger sibling of eight years showing diffuse swelling over the hard palate and multiple unerupted teeth

**Figure 4:**
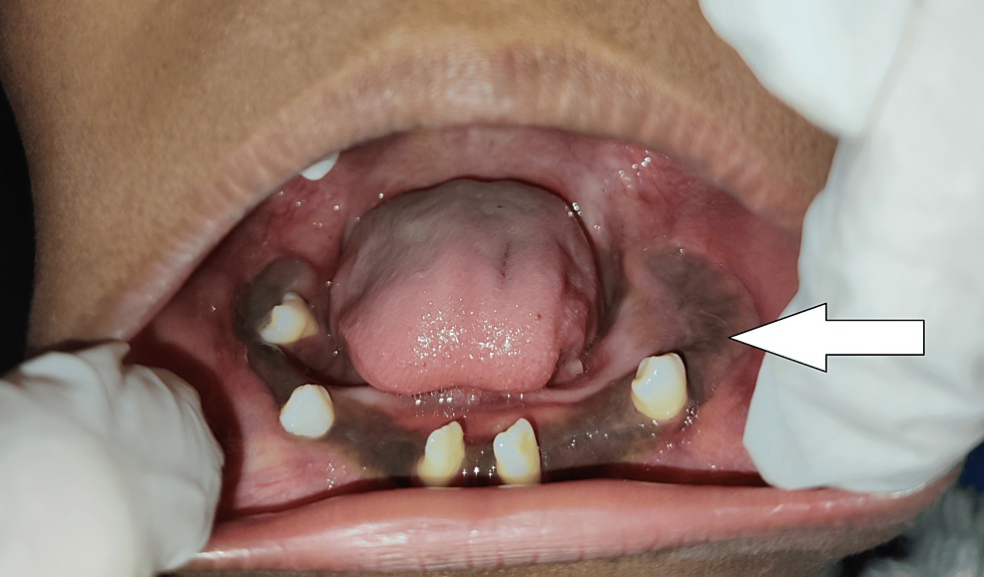
Patient 1 (younger sibling of eight years) showing cortical expansion and multiple unerupted teeth

**Figure 5:**
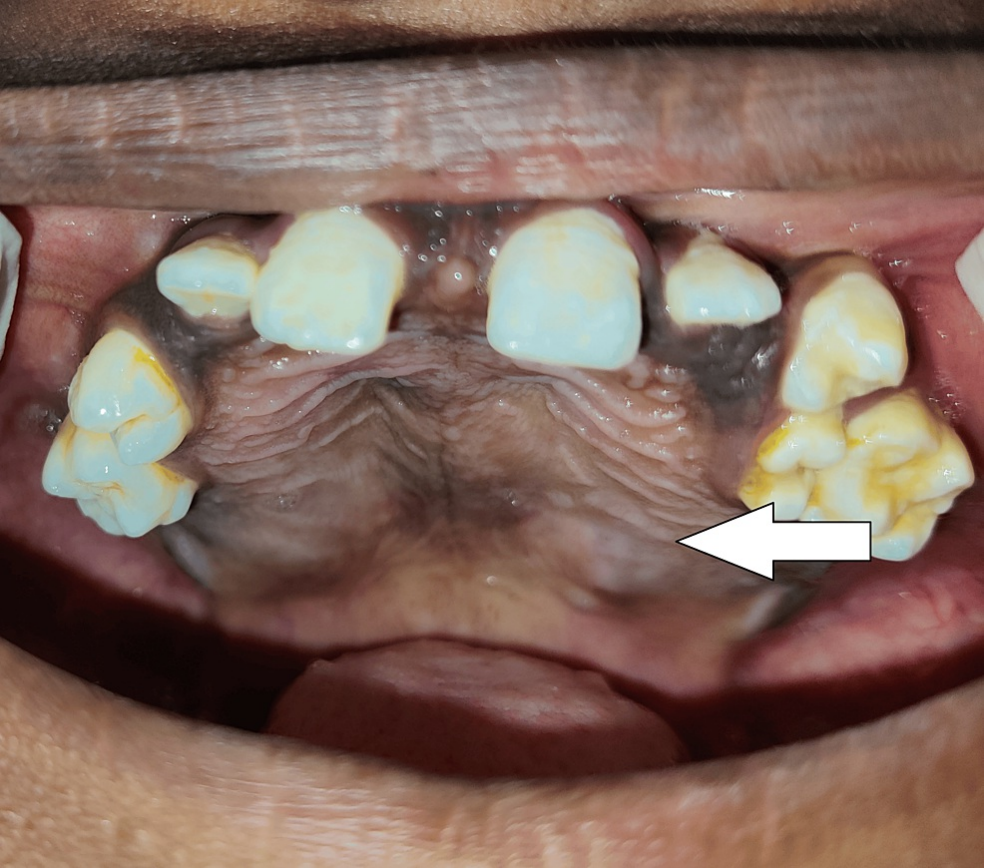
Patient 2 (older sibling of 10 years) showing diffuse swelling over the hard palate and multiple malposed teeth

**Figure 6:**
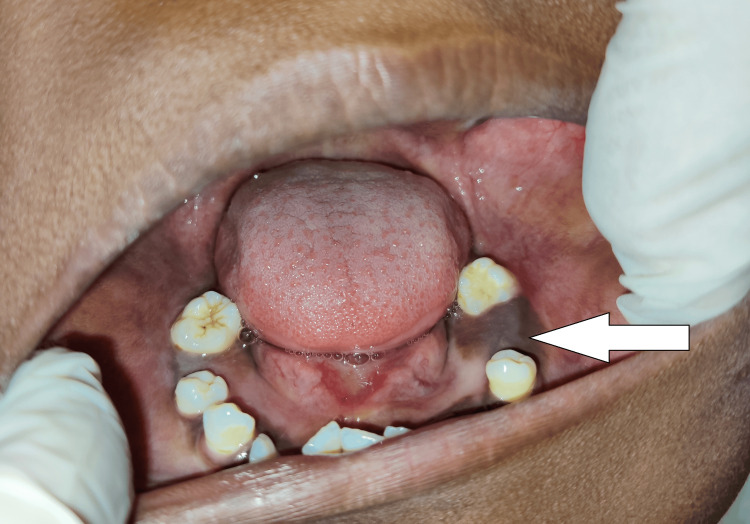
Patient 2 (older sibling of 10 years) showing cortical expansion and multiple unerupted teeth

A few permanent teeth were visible and others were absent during the intraoral examination, in both the siblings. Expansion of cortical plates could be seen in both patients. There was a diffuse swelling involving the palate and the floor of the mouth in both siblings (Figures 3-6).

Blood chemistry was performed to rule out fibrous dysplasia and hyperparathyroidism. Laboratory investigation of the younger sibling revealed alkaline phosphatase within normal limits, i.e., 266 units per liter (U/L) (normal 100-250 U/L), serum sodium level was 141.5 miliequivalent per liter (mEq/L) (normal 128-148 mEq/L), serum potassium 3.74 mEq/L (normal 3.5-5.5 mEq/L), total calcium level was 9.80 milligrams per deciliter (mg/dL) (normal 8.7-10.8 mg/dL), and ionic calcium was 1.22 mg/dL (normal 1.10-1.35 mg/dL). Whereas the laboratory investigation of the elder sibling revealed alkaline phosphatase within normal limits 201 U/L, serum sodium was 141.2 mEq/L, serum potassium was 3.56 mEq/L, total calcium level was elevated to 11.25 mg/dL, and ionic calcium was also elevated to 1.40 mg/dL.

**Figure 7:**
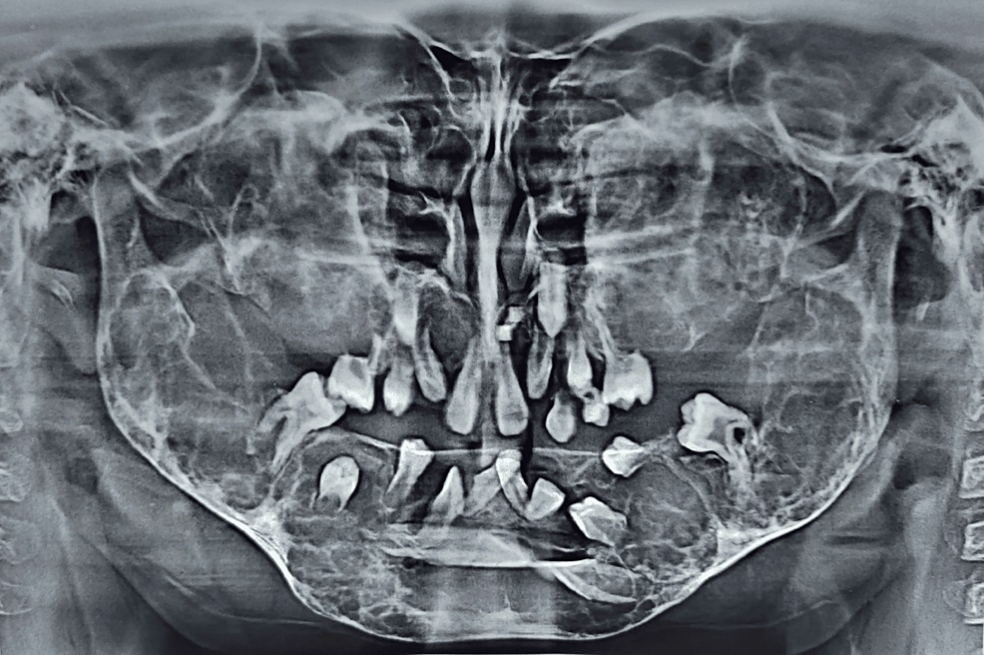
(Patient 1: 8 years old) OPG taken at the age of six years showing multiple multilocular radiolucencies all over the maxilla and mandible and multiple impacted and displaced teeth OPG: orthopantomogram

**Figure 8:**
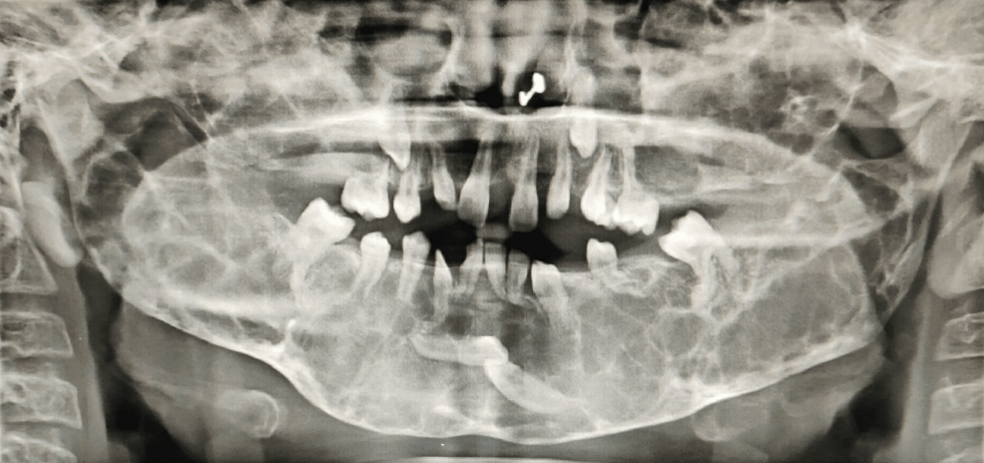
(Patient 1: 8 years old) OPG taken at the age of eight years showing multiple multilocular radiolucencies all over the maxilla and mandible and multiple impacted and displaced teeth OPG: Orthopantomogram

**Figure 9:**
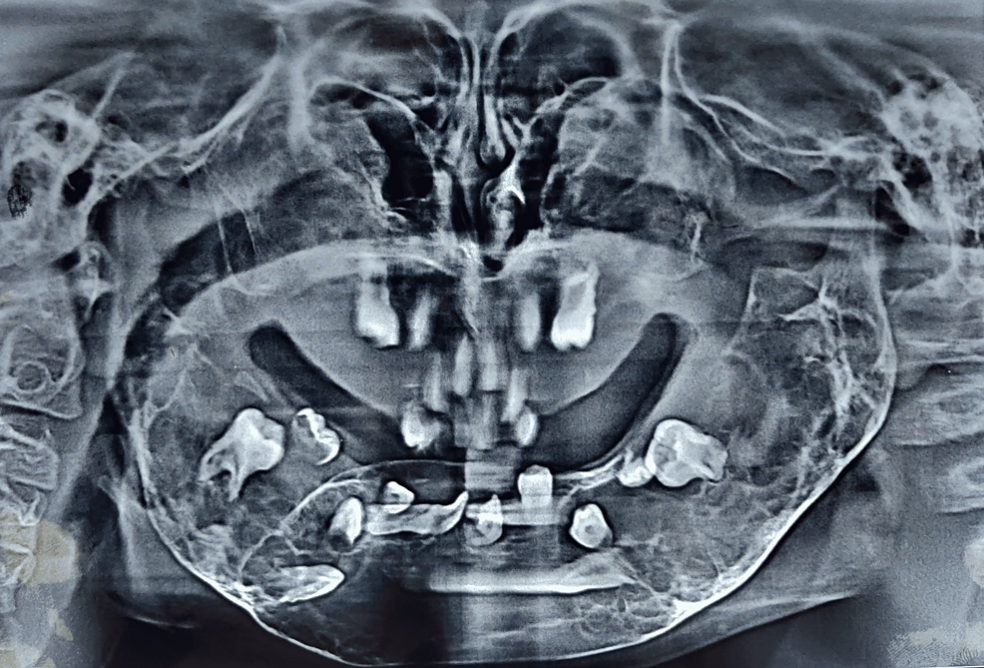
(Patient 2: 10 years) OPG taken at the age of eight years showing multiple multilocular radiolucencies in the whole mandible and multiple impacted and displaced teeth in the upper and lower jaws OPG: Orthopantomogram

**Figure 10:**
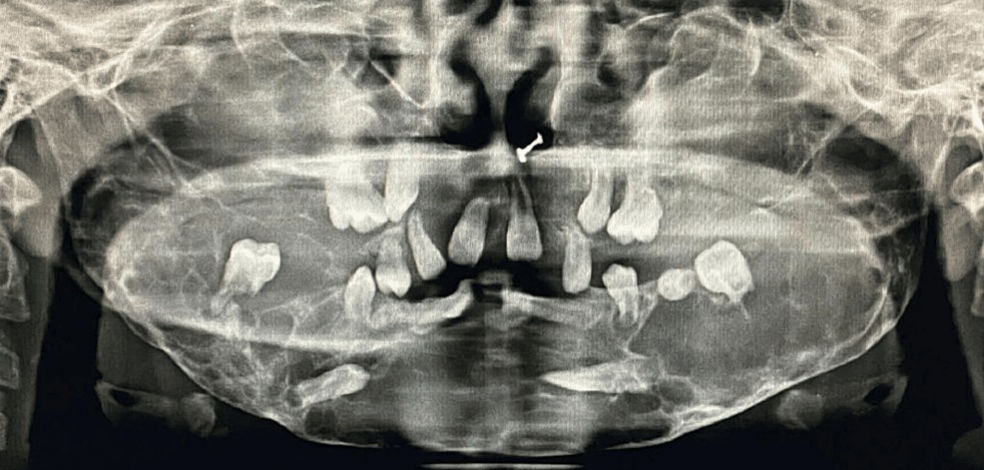
(Patient 2: 10 years) OPG taken at the age of 10 years showing multiple multilocular radiolucencies in the whole maxilla and mandible with multiple impacted and displaced teeth OPG: Orthopantomogram

Panoramic radiographs of both siblings show multiple well-defined multilocular lesions which were bilaterally symmetrical in the whole mandible and maxilla. Multiple impacted and displaced teeth can be seen in the panoramic view (Figures 7-10).

**Figure 11:**
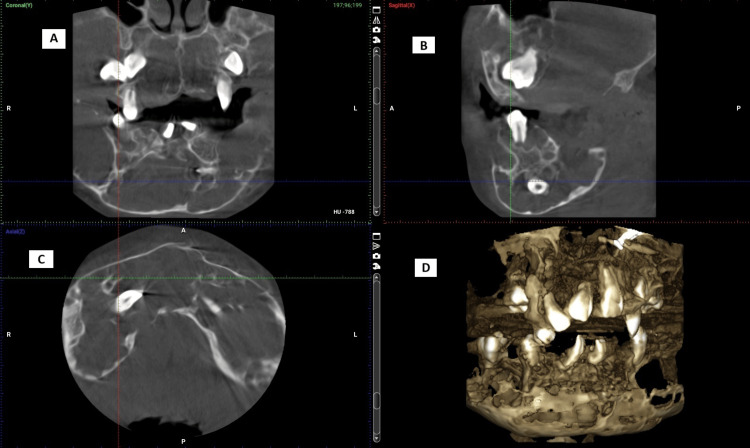
(Patient 1: 8 years) CBCT findings revealing multiple multilocular lesions in the maxilla and the mandible with impacted and displaced teeth. It also shows thin, coarse trabecular pattern of bone in the maxilla and the mandible CBCT: Cone-beam computed tomography Panel A: showing the coronal view on CBCT revealing impacted 13 and 23 on coarse trabecular pattern of the bone in the maxilla and the mandible; Panel B: showing the sagittal view on CBCT revealing fine and thin trabecular patterns ofthe bone in  the maxilla and the mandible; Panel C: showing the axial view on CBCT revealing buccal and lingual cortical expansion with thinning of the cortical boundaries; Panel D: showing a 3D construction on CBCT revealing multiple multilocular lesions in the maxilla and the mandible with impacted and displaced teeth

**Figure 12:**
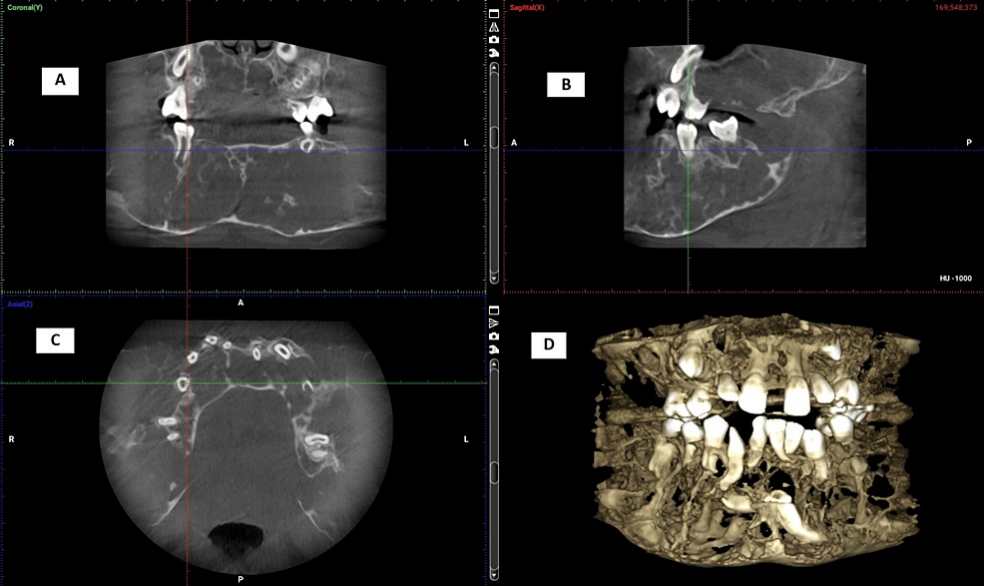
(Patient 2: 10 years) CBCT findings revealing multiple multilocular lesions in the maxilla and the mandible with impacted and displaced teeth. It also shows thin, coarse trabecular pattern of the bone in the maxilla and the mandible CBCT: Cone-beam computed tomography Panel A: showing the coronal view on CBCT revealing impacted 13 and 23 on coarse trabecular pattern of the bone in the maxilla and the mandible; Panel B: showing the sagittal view on CBCT revealing fine and thin trabecular patterns of the bone in the maxilla and the mandible; Panel C: showing the axial view on CBCT revealing buccal and lingual cortical expansions with thinning of cortical boundaries; Panel D: showing a 3D construction on CBCT revealing multiple multilocular lesions in the maxilla and the mandible with impacted and displaced teeth

The cone-beam computed tomography (CBCT) findings show multiple, well-defined multilocular radiolucent lesions involving the whole maxilla and the mandible. The multilocular lesions are of varied sizes with fine trabecular patterns giving it a soap-bubble appearance. With the expansion of the lingual and buccal cortical plates of the upper and lower jaws with thinning of the cortex, multiple displaced and impacted teeth can be seen in the maxilla as well as the mandible. The erupted teeth are displaced and exhibit a floating tooth appearance (Figure 11 and Figure 12).

Cherubism was the diagnosis given for both siblings based on radiographic and blood chemistry findings. The differential diagnoses considered for the case are polyostotic fibrous dysplasia, giant cell granuloma of jaw, and osteoclastoma. As it is a self-limiting condition that was associated with no functional disability, hence, no medical care was done. Further surgical treatment is postponed until the sibling’s reached puberty, if required. The parents of the siblings were educated about the condition and were advised to bring the children for a periodic follow-up. 

## Discussion

Cherubism is categorized by the World Health Organization as a kind of nonneoplastic bone lesion that affects the maxilla and the mandible [1]. Known to be a benign, infrequent condition with autosomal dominant inheritance, it is one of the very rare human osteoclastic lesions that is dictated by genetics. The disorder has been referred to by several names, such as bilateral giant cell tumor, familial multilocular disease, and hereditary or familial fibrous dysplasia [2].

Male prevalence is 100% compared to female prevalence, which is 50-70%, or a 2:1 ratio. Cherubism is caused by chromosome 4p16.3, which is located between 4p-telomere and D4S127, which is known as SH3-binding protein (2SH3BP2) [3,4]. Root resorption, impacted and irregularly shaped teeth, tooth exfoliation, tooth malposition, agenesis of permanent teeth, and ectopic eruption, are dental anomalies associated with this condition. These anomalies cause phonation and swallowing issues, as well as malocclusion. These anomalies, which result in occlusal conditions, are caused by the replacement of fibrous tissue by bone [5]. 

Cherubism does not show up clinically or radiographically until a child is between 14 months and 3 years old. Typically, a lesion advances more quickly the sooner it manifests. In all cases, the face gradually swells, with the cheeks and jaws becoming noticeably fuller; the skin and subcutaneous tissue remain normal. This swelling is caused by the underlying skeletal structures growing larger and expanding. When present, the bilateral expansion of the maxilla helps a cherubic image by extending the cheek and exposing a thin line of sclera, giving the appearance that the eyes are elevated to heaven [6].

This condition is distinguished radiographically by bilateral multilocular cystic enlargement of the jaws. The posterior body of the mandible and the ascending rami both experience early lesions [7]. Because the sinus and nasal cavities overlap, maxillary lesions may develop concurrently yet go undetected on radiographs [7,8]. There have been reports of the inferior alveolar canal being displaced. The teeth may shift as a result of the deterioration of the alveolar cavity, creating a radiological image known as "floating tooth syndrome." Cystic portions of the jawbone ossify as people age, resulting in patchy, atypical sclerosis. The tiny, compressed trabecular pattern has a typical (but nonspecific) ground-glass appearance [9].

Cherubism-related syndromes include Ramon syndrome, Noonan syndrome, and neurofibromatosis type 1. Differential diagnosis includes myxoma, bilateral parotid swelling, giant cell recurrent granuloma, giant cell tumor, ameloblastoma, bilateral odontogenic cysts, histiocytosis X (Hand-Schuller-Christian disease), odontogenic keratocyst (including nevoid basal cell tumor syndrome), Jaffe-Campanacci syndrome, Brown tumor (hyperparathyroidism), infantile cortical hyperostosis, fibro-osteoma, ossified fibroma, fibrous dysplasia, odontogenic fibroma, and aneurysmal cysts [10,11]. While alkaline phosphatase levels are increased, biochemical tests can reveal normal ranges for blood follicle-stimulating hormone, phosphorus and calcium concentrations, luteinizing hormone, T4 and T3 levels, and thyroid-stimulating hormone. Histologically, the lesion appears as a smattering of multiple multinucleated large cells inside a fibrous connective tissue [5].

Ongole et al. in 2003 reported a case of siblings of a girl and a boy with slowly growing bilateral swelling of the jaws with panaromic finding of bilateral radiolucencies extending over the body and ramus of the mandible; however, the condyles were unaffected and the diagnosis of the case was given as cherubism [1]. Muthuraman et al. in 2014 described a rare hereditary case of cherubism which was conservatively treated [5]. Cherubism often has a positive outlook as after puberty, and the complete lesion tends to take a normal appearance as the patient matures and achieves adulthood and does not progress. Surgery is not a preferred form of therapy. However, if tissue enlargement leads to trouble with the airway or chewing capacity, it is possible to do surgery and biopsies. Concerns about appearance and functionality necessitate medical care.

## Conclusions

The clinical and radiographic findings of both siblings in this case confirm most other reported cases of cherubism. Laboratory evaluations on blood alkaline phosphatase, phosphate, and calcium are required to distinguish this condition from other similar lesions. Until the lesions develop any functional difficulties, patients can merely undertake periodic examinations to watch for aggressive transformation or systemic involvement, since most such lesions disappear with age. Nonetheless, surgical intervention involving debulking of lesions and surgical recontouring may be considered in individuals with functional, cosmetic, or emotional issues.

## References

[1] Ongole R, Pillai RS, Pai KM (2003). Cherubism in siblings: a case report. J Can Dent Assoc.

[2] Degala S, Mahesh KP, Monalisha Monalisha (2015). Cherubism: a case report. J Maxillofac Oral Surg.

[3] Morice A, Joly A, Ricquebourg M (2020). Cherubism as a systemic skeletal disease: evidence from an aggressive case. BMC Musculoskelet Disord.

[4] Dinca O, Severin E, Vladan C, Bodnar DC, Bucur A (2014). Cherubism: a case report. Rom J Morphol Embryol.

[5] Muthuraman V, Srinivasan S (2014). Familial case of cherubism from South India: differential diagnosis and report of 2 cases. Case Rep Dent.

[6] Sidorowicz W, Kubasiewicz-Ross P, Dominiak M (2018). Familial cherubism: clinical and radiological features. Case report and review of the literature. Eur J Paediatr Dent.

[7] Goyal V, Jasuja P (2009). Cherubism: a case report. Int J Clin Pediatr Dent.

[8] Kaur M, Shah S, Babaji P, Singh J, Nair D, Kamble SS (2014). Cherubism: a rare case report. J Nat Sci Biol Med.

[9] Jung KW, Yun JM, Lee JM, Choi IS (2022). Sinonasal manifestations of severe cherubism: a case with 11-year follow-up. Ear Nose Throat J.

[10] Tekin AF, Ünal ÖF, Göksel S, Özcan İ (2020). Clinical and radiological evaluation of cherubism: a rare case report. Radiol Case Rep.

[11] Lahfidi A, Traore WM, Diallo ID, Lrhorfi N, Elhaddad S, Allali N, Chat L (2022). Cherubism: a rare case report with literature review. Radiol Case Rep.

